# Contributors to the *RSC Chemical Biology* Emerging Investigators Collection 2024

**DOI:** 10.1039/d5cb90017d

**Published:** 2025-04-11

## Abstract

This article profiles the early career researchers whose work features in the *RSC Chemical Biology* Emerging Investigators Collection 2024.
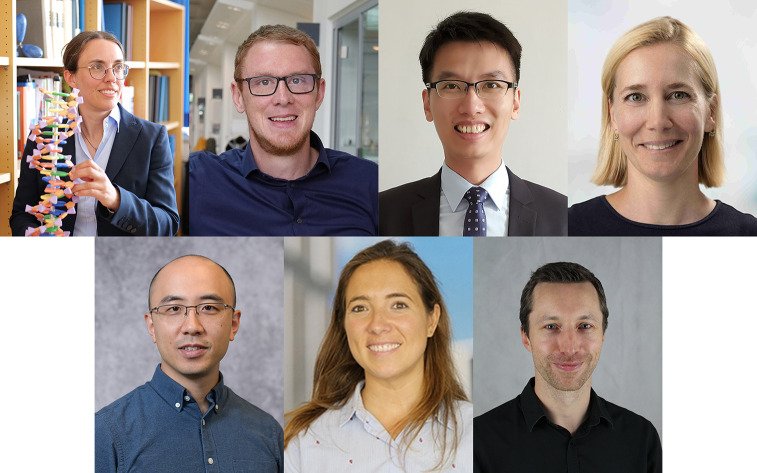


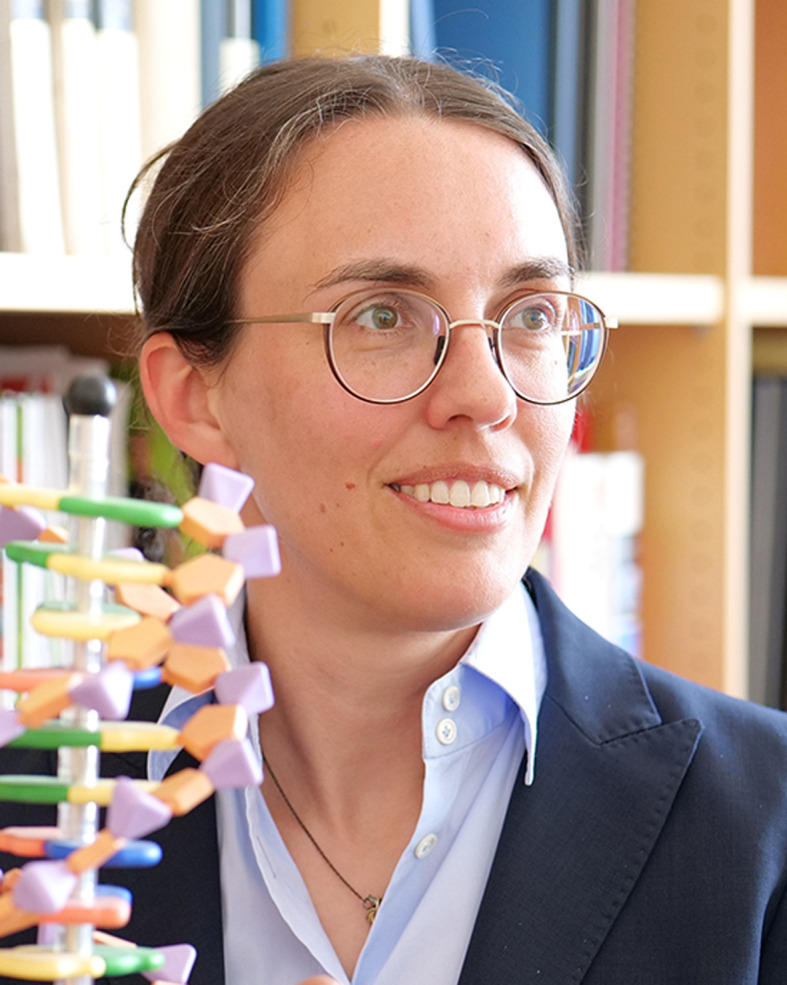
Stephanie Kath-Schorr is a professor of organic chemistry at the University of Cologne. Her research focuses on the field of nucleic acid chemistry and chemical biology with an emphasis on the synthesis and investigation of chemically modified nucleic acids. Kath-Schorr received her PhD in 2010 from the Ludwig-Maximilians-Universität in Munich, where she worked on DNA damage caused by aromatic amino compounds. After a post-doctoral period at the University of Dundee in Scotland, she returned to Germany in 2013 to establish a junior research group in chemical biology at the LIMES (Life and Medical Sciences) Institute at the University of Bonn, funded by the Fonds der Chemischen Industrie, before accepting a professorship at the University of Cologne in 2020.

Her contribution to the 2024 *RSC Chemical Biology* Emerging Investigators collection can be read at https://doi.org/10.1039/D4CB00084F.
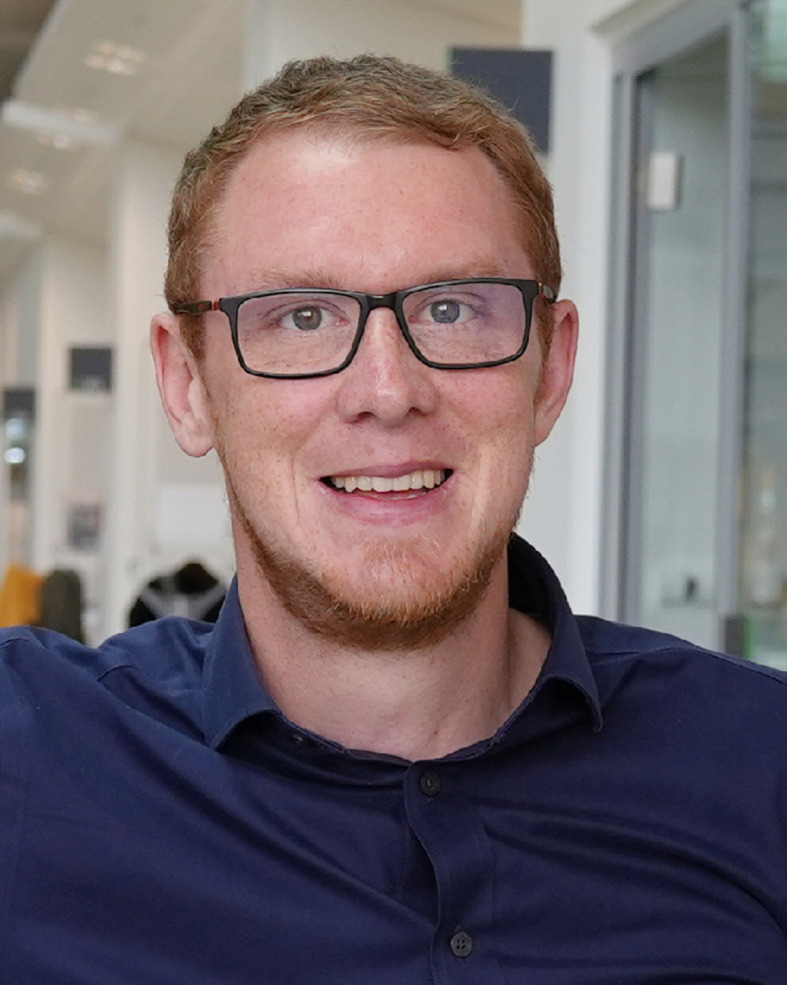


Ben Schumann trained in carbohydrate chemistry with Peter Seeberger at the Max Planck Institute in Potsdam, and in chemical glycobiology with Prof. Carolyn Bertozzi at Stanford. Combined with a background in biochemistry, he has learnt to use synthetic tools to probe, understand and manipulate glycans, particularly in the secretory pathway of mammalian cells. A breakthrough technology in his lab features the use of engineered glycosyltransferases and biosynthetic enzymes to generate “precision tools” for individual enzymes, glycan sub-types and cells. Ben has received multiple awards including a Chemical Biology Horizon Prize (2021), the Dextra (2023) and Heatley (2024) awards from the Royal Society of Chemistry.

His contribution to the 2024 *RSC Chemical Biology* Emerging Investigators collection can be read at https://doi.org/10.1039/D4CB00093E.
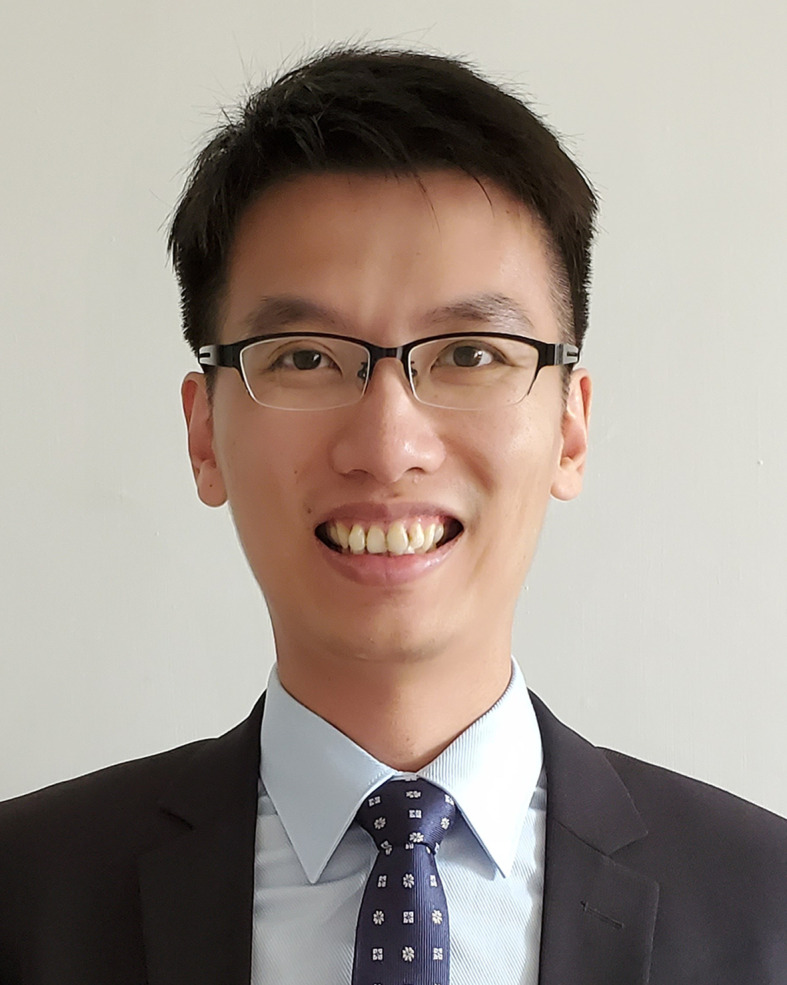


Clive Yik-Sham Chung is an assistant professor in the School of Biomedical Sciences and Department of Pathology at The University of Hong Kong (HKU). He obtained his BSc in chemistry and PhD under the supervision of Prof. Vivian Yam at HKU. He then conducted postdoctoral studies with Prof. Chi-Ming Che on inorganic medicine, Prof. Christopher Chang on molecular imaging and Prof. Daniel Nomura on chemoproteomics. In 2020, he established his own laboratory at HKU, focusing on the development of: (1) chemoproteomics probes to identify new druggable hotspots; (2) molecular tools for investigating cellular redox biology; (3) novel therapeutic covalent ligands for targeted therapy.

His contribution to the 2024 *RSC Chemical Biology* Emerging Investigators collection can be read at https://doi.org/10.1039/D4CB00111G.
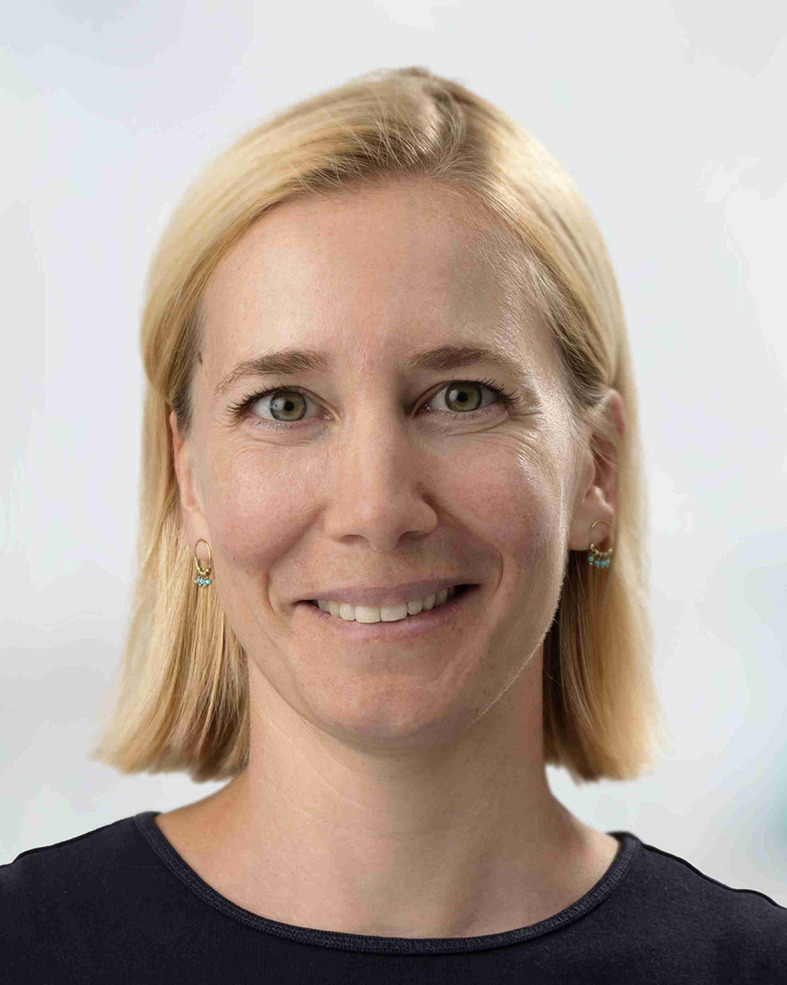


Claudia Jessen-Trefzer is a group leader at the University of Freiburg in Germany. She earned her Diploma in chemistry from the University of Konstanz and her PhD in chemistry from the Swiss Federal Institute of Technology in Lausanne. With a long-standing fascination for mycobacteria, she has dedicated the past few years to investigating mycobacterial protein nanocapsids and their applications in organometallic catalysis. Under her leadership, her research group is pioneering the use of encapsulation techniques to advance bioorthogonal chemistry, as well as exploring innovative solutions for prodrug activation and targeted cell therapies.

Her contribution to the 2024 *RSC Chemical Biology* Emerging Investigators collection can be read at https://doi.org/10.1039/D4CB00127C.
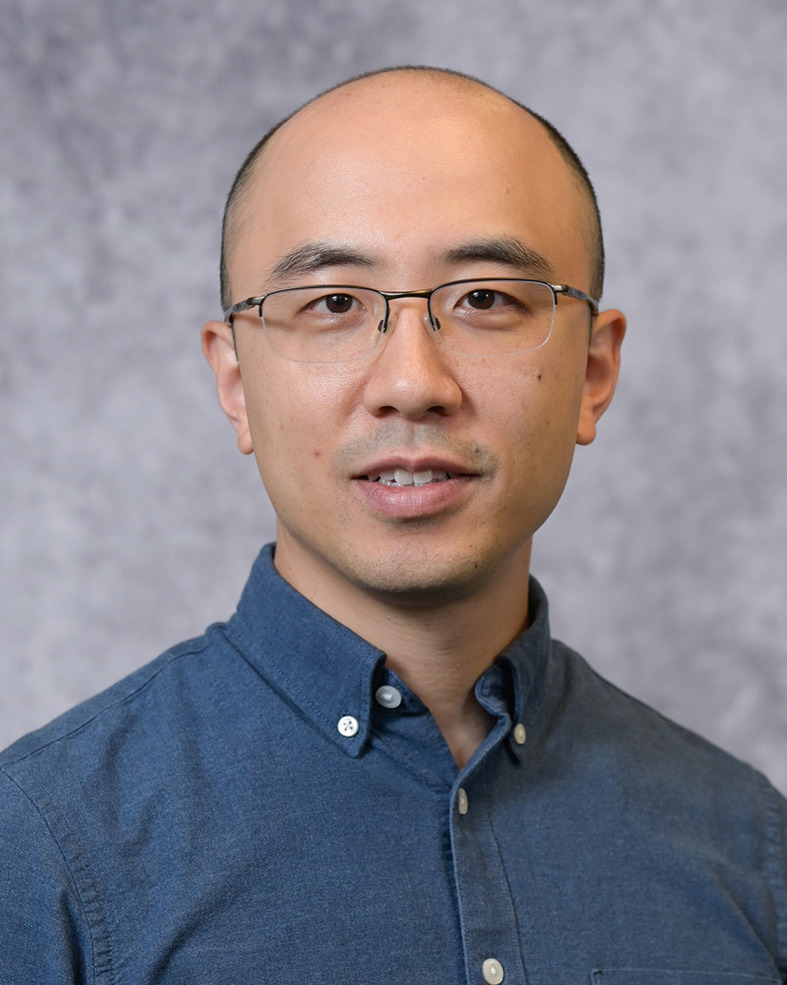


Jun Ohata was born and raised in Japan. He received his BSc and MSc from Osaka Prefecture University, where he worked with Prof. Matsuzaka studying the carbon species on diruthenium complexes. He earned his PhD in the Ball group at Rice University, focusing on metal-catalyzed protein bioconjugation. He conducted his postdoctoral work with Prof. Chang at the University of California–Berkeley as a JSPS Postdoctoral Fellow, developing chemical probes for the detection of cellular metal ions. He then took up his current position at North Carolina State University as an assistant professor developing novel strategies for bioconjugation.

His contribution to the 2024 *RSC Chemical Biology* Emerging Investigators collection can be read at https://doi.org/10.1039/D4CB00142G.
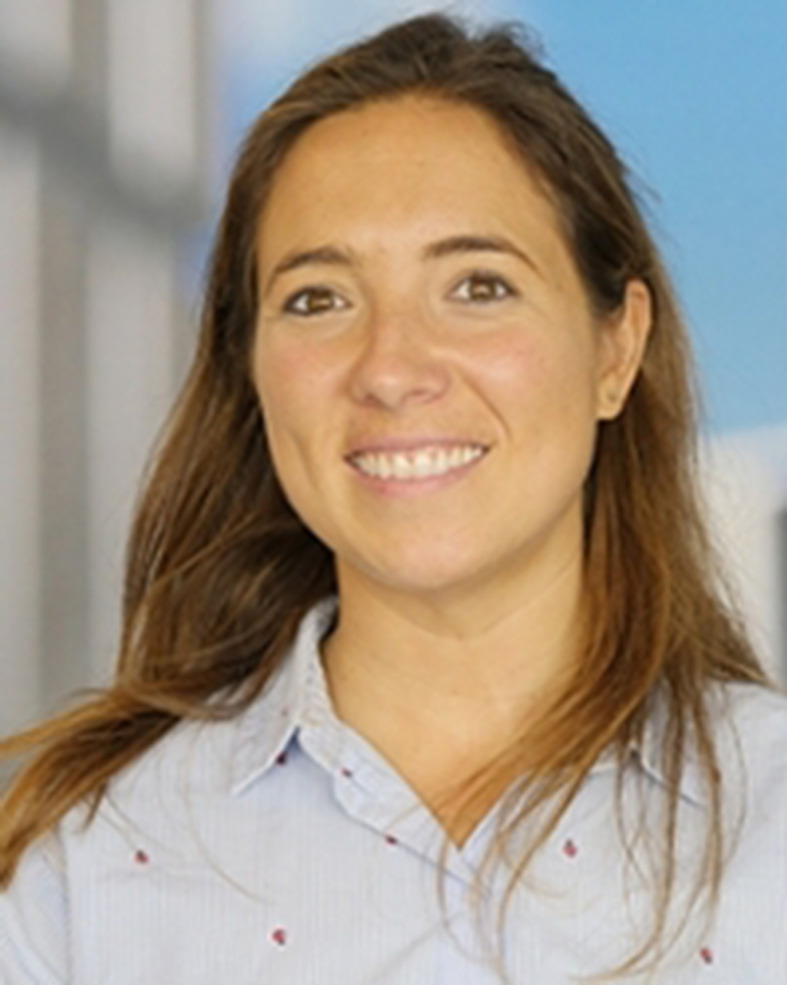


Marta Artola is an assistant professor and group leader at the Medical Biochemistry Department of the Leiden Institute of Chemistry (LIC), Leiden University, where she combines organic chemistry, chemical biology, and medical glycobiology. Her research focuses on developing chemical biology tools to study and manipulate glycoprocessing enzymes (CAZymes) for therapeutic applications, including enzyme inhibitors, chaperones, protein degraders, and activity-based probes. Artola earned her PhD in organic chemistry in 2014 from the Universidad Complutense de Madrid, complemented by research stays at The Scripps Research Institute and the Technische Universität München. After postdoctoral research at Leiden University with Prof. Herman Overkleeft, she launched her independent lab in 2019, supported by a recently awarded ERC Starting Grant.

Her contribution to the 2024 *RSC Chemical Biology* Emerging Investigators collection can be read at https://doi.org/10.1039/D4CB00218K.
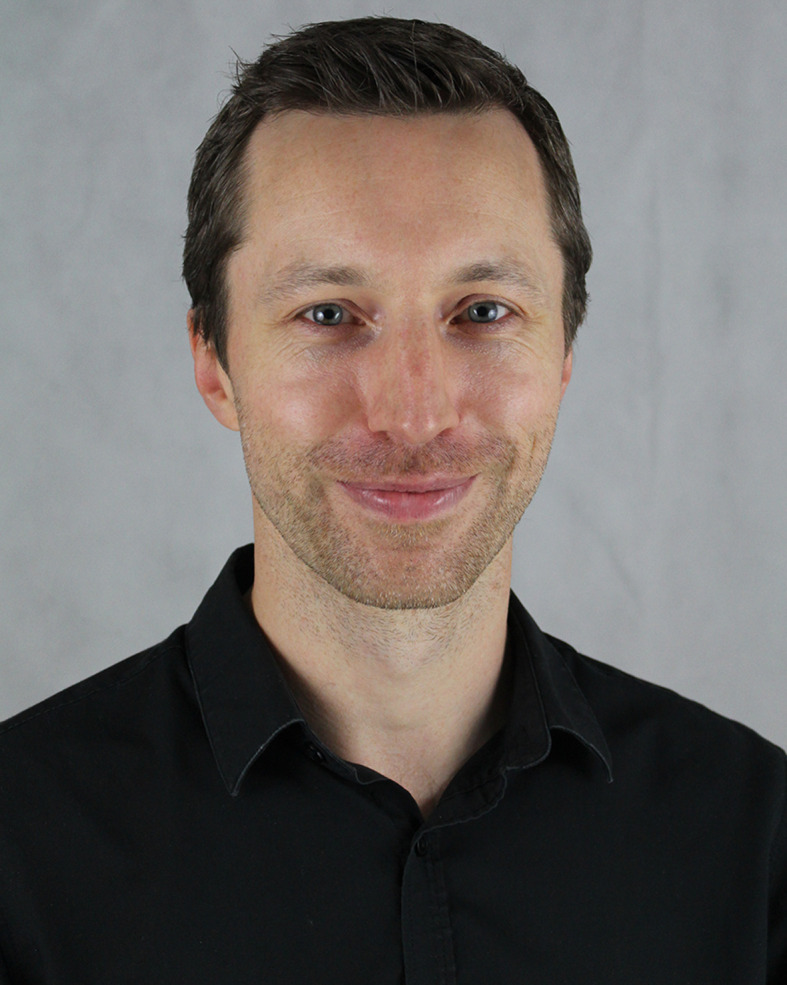


Tom Lanyon-Hogg is a Career Development Fellow at the University of Oxford in the Department of Pharmacology. He completed his Master’s in medicinal chemistry and PhD in biochemistry at the University of Leeds, before joining the group of Prof. Ed Tate at Imperial College London as a postdoctoral researcher in chemical biology. In 2020 Tom joined the University of Oxford to start his independent career. The Lanyon-Hogg group’s research focuses on the development of chemical tools to identify and validate new targets to combat antimicrobial resistance.

His contribution to the 2024 RSC Chemical Biology Emerging Investigators collection can be read at https://doi.org/10.1039/D4CB00291A.

